# Isolation of nanobodies against *Xenopus* embryonic antigens using immune and non-immune phage display libraries

**DOI:** 10.1371/journal.pone.0216083

**Published:** 2019-05-02

**Authors:** Keiji Itoh, Alice H. Reis, Andrew Hayhurst, Sergei Y. Sokol

**Affiliations:** 1 Department of Cell, Developmental and Regenerative Biology, Icahn School of Medicine at Mount Sinai, New York, NY, United States of America; 2 Department of Virology and Immunology, Texas Biomedical Research Institute, San Antonio, TX, United States of America; University of Colorado Boulder, UNITED STATES

## Abstract

The use of *Xenopus laevis* as a model for vertebrate developmental biology is limited by a lack of antibodies specific for embryonic antigens. This study evaluated the use of immune and non-immune phage display libraries for the isolation of single domain antibodies, or nanobodies, with specificities for *Xenopus* embryonic antigens. The immune nanobody library was derived from peripheral blood lymphocyte RNA obtained from a llama immunized with *Xenopus* gastrula homogenates. Screening this library by immunostaining of embryonic tissues with pooled periplasmic material and sib-selection led to the isolation of several monoclonal phages reactive with the cytoplasm and nuclei of gastrula cells. One antigen recognized by a group of nanobodies was identified using a reverse proteomics approach as nucleoplasmin, an abundant histone chaperone. As an alternative strategy, a semi-synthetic non-immune llama nanobody phage display library was panned on highly purified *Xenopus* proteins. This proof-of-principle approach isolated monoclonal nanobodies that specifically bind Nuclear distribution element-like 1 (Ndel1) in multiple immunoassays. Our results suggest that immune and non-immune phage display screens on crude and purified embryonic antigens can efficiently identify nanobodies useful to the *Xenopus* developmental biology community.

## Introduction

For several decades, *Xenopus laevis* embryos have been a leading non-mammalian model for vertebrate embryology. Major advances have been made using this model, including the discoveries of nuclear reprogramming [1], localized maternal RNAs [2], key cell cycle components [3] and signaling factors mediating mesoderm and neural tissue induction [4–8]. Despite these achievements, lack of antibodies specific to embryo components remains a major challenge impeding further progress of molecular and cell biological studies using *Xenopus*.

To address this issue, we compared different approaches for the rapid isolation of multiple recombinant antibodies that specifically react with *Xenopus* embryonic antigens. Naturally occurring single domain antibodies (or nanobodies) of camelids are especially useful, because of their exceptional stability *in vitro* and *in vivo* when they are expressed in living cells [9–14]. Moreover, nanobodies can be directly obtained from the periplasmic compartment of recombinant bacteria, readily stored protein and immortalized as DNA or *in silico* [15–17]. Herein, we describe the use of immune and non-immune phage display libraries for the isolation of several nanobodies specific for a variety of *Xenopus* antigens.

## Materials and methods

### Ethics statement

This study was carried out in strict accordance with the recommendations in the Guide for the Care and Use of Laboratory Animals of the National Institutes of Health. The protocol 04–1295 was approved by the IACUC of the Icahn School of Medicine at Mount Sinai.

### Xenopus embryos

Eggs were obtained from *Xenopus laevis* females (NASCO) after injection of 700–800 units of human chorionic gonadotropin. Frog handling was according to the animal protocol approved by the MSSM IACUC. In vitro fertilization, embryo culture in 0.1 x Marc’s modified Ringer (MMR) solution and staging were carried out as described [18–20]. Embryos were injected at the 4-cell stage with 100 pg of Ndel1 plasmid (25 pg per injection in each blastomere) and were cultured until stage 22 or 38 when they were lysed in 1% Triton X100, 50 mM NaCl, 1 mM EDTA and 10 mM Tris HCl (pH 7.5).

### Construction of a nanobody phage display library from an immune llama

To prepare immunogen, a mixture of *Xenopus laevis* gastrula and neurula embryos was homogenized in 0.1 x MMR by pipetting and fractionated by centrifugation at 1800 g for 15 min. Several layers became visible after the fractionation, including yolk platelets, ‘pigmented and clear fractions’ and the lipid layer (from bottom to top). Whole blood was obtained from a llama (Triple J Farm, WA) immunized six times at 3-week intervals with the clear fraction of (approx. 200 micrograms of protein per injection), which contains cytoplasm, membranes as well as nuclei, as previously described [21]. The blood diluted with PBS was layered on top of Lympholyte (Cedarlane) and centrifuged at 800 g for 20 min to collect white blood cells at the interface. Total RNA was extracted from white blood cells using RNAeasy miniprep kit (Qiagen). 18 μg of total RNA was obtained from 5 x 10^7^ cells and used to synthesize cDNA with first strand cDNA synthesis kit (Superscript II, Invitrogen), following manufacturer’s instructions. DNA fragments corresponding to variable heavy chain (VHH or nanobody) were amplified from the cDNA with Pfu DNA polymerase and nested primers designed as described [11, 22] with modifications. The first PCR was performed with the following two sets of primers.

Ryc-Fw 1, 5’ GTCCTGGCTGCTCTTCTACAAGG 3’ and

Ryc-Rv 1, 5’ GGTACGTGCTGTTGAACTGTTCC 3’;

Lad-Fw 1, 5’ GAK GTS CAG CTG CAG GCG TCT GGR GGA GG 3’ and

Lad-Rv 1, 5’ CGC CAT CAA GGT ACC AGT TGA 3’.

The PCR products (600–800 bp) were gel purified and subjected to a second nested PCR with two sets of primers as follows. Ryc-Fw 3, 5’ ATG GCC CAG CCG GCC GTG CAG CTG GTG GAG TCT G 3’ and Ryc-Rv 2, 5’ TTA TGC GGC CGC CGA GGA GAC GGT GAC CTG GGT 3’; Lad-Fw 3, 5’ ATG GCC CAG CCG GCC GAK GTS CAG CTG CAG GCG 3’ and Lad-Rv-2, 5’ AT TGC GGC CGC TGA GGA GAC GGT GAC CTG 3’. Final PCR products of 350–450 bp (50 μg) and the phage display vector, pecan126 (48 μg) that endows sdAb with biotinylation acceptor peptide, His^6^-tag, and optional M13 phage gene 3 fusion [23] were digested with *Sfi* I and *Not* I, treated with calf intestinal phosphatase CIP, gel purified and ligated with T4 DNA ligase. The ligated material (300 μl) was extracted with phenol/chloroform and chloroform, ethanol precipitated and dissolved in 60–120 μl H_2_O for electroporation into HBV88 cells.

HBV88 cells contain a chloramphenicol-resistant plasmid encoding a suppressor tRNA which is expressed upon arabinose induction [23]. The cells were made electrocompetent using low salt YENB media followed by extensive washing as described [23]. Bacteria were streaked on M9/chloramphenicol (Cm) + thiamine M9 minimal agar to select for the F’-episome. A single colony was cultured first in 2 ml of liquid M9 media for 8 h. 200 μl of the culture was added to 2 x 50 ml liquid M9 and shaken overnight at 37^0^ C. 10 ml of the culture was added to 6 x 400 ml flasks of YENB plus Cm to OD_600_ = 0.05 and shaken at 30^0^ C until OD = 0.6 (approx. 5 h). After chilling on ice, the cultures were pelleted in Sorvall RC3C+ centrifuge at 4000 g at 4°C for 20 min. Each pellet from 6 preparations was resuspended gently in 50 ml ice-cold water and transferred into 2 x 50 ml conical tubes. Ice-cold water was added to 50 ml and mixed. Cells were pelleted at 4000 g at 4°C for 15 min. This washing procedure was repeated. Cells were further washed in 25 ml of ice-cold 15% glycerol, pelleted again and resuspended in 8.7 ml of 15% glycerol. Competent cells were aliquoted at 270 μl into Eppendorf tubes (pre-chilled), frozen in dry ice and stored at -80°C. Transformation efficiency was 5.2 x 10^8^ CFU/μg with pecan126 plasmid.

For electroporation using Gene Pulser (Bio-Rad), 60–90 μl of the ligated material and 270 μl of electrocompetent HVB88 cells were mixed, placed into a 2 mm-gap cuvette (BioRad or Bulldog Bio) and received a 2.5 kV pulse at 25 μF and 200 Ω. The electroporated cells were added to 2 ml of warm (37°C) super optimal broth (SOB) + 2% glucose in a 15 ml tube and incubated on a platform shaker at 37 ^0^C for 1 h. The culture was centrifuged at 3000 rpm for 10 min. The pellets were resuspended in 500 μl SOB+2% glucose and each suspension was spread onto two 15 cm 2 x YT + 2% glucose plates (Cm and 200 μg/ml ampicillin [Amp])). The plates were incubated at 37°C overnight. Approximately 10^8^ transformants were obtained with the transformation efficiency of 2x10^6^ CFU/μg. All colonies were collected in 70 ml of 2 x YT + 2% glucose media and an equal volume (70 ml) of ice cold 30% glycerol in Terrific Broth was added and mixed well. 1.3 ml aliquots in Eppendorf tubes were frozen on dry ice and stored at -80°C.

The pecan126 library was rescued as described [23]. 430 μl of the thawed HBV88 library stock were added to 500 ml 2x YT 2% glucose (Amp, Cm) to make an OD_600_ = 0.05, and cultured until OD_600_ = 0.44 was reached (approximately 2 h at 37°C). Helper phage M13K07 was added at a multiplicity of infection (MOI) of 20 to the culture and incubated static at 37 ^0^C for 30 min and then shaken at 200 rpm for 30 min at 37 ^o^C. L-Arabinose (2000 μg/ml, final), IPTG (10 μM) and kanamycin (70 μg/ml) were added to the culture, which was shaken at 30 ^0^ C for 22 h. The culture was transferred to 16 round bottom 30 ml tubes (for high speed centrifugation) and centrifuged with Sorvall 5C Plus at 7700 g for 1 h at 4 ^0^C. 100 ml of 20% PEG8000+ 2.5 M NaCl were added to the supernatant and stirred at 4 ^0^C overnight. 600 ml of the phage were centrifuged at 4100 g for 1 h at 4 ^0^C. The pellets were resuspended in 3.3 ml of PBS, mixed with 3.3 ml of glycerol and aliquoted at 500 μl for storage at -80^0^ C. The titer of the phage library was 3.6 x 10^10^ cfu/ml. The nanobody DNA sequences reported in this paper are presented in S1 Fig.

### Phage display screen using the immune llama cDNA library

Ten to twenty embryos at stage 11 and 14 were fixed with 3.7% formaldehyde in PBS for 1 h and subsequently with Dent’s fixative (80% methanol and 20% DMSO) for 1 h. The fixed embryos were washed with PBS three times and homogenized with a pestle after adding 50 μl PBS in 1.5 ml Eppendorf tubes. 2% skim milk in PBS (1 ml) was added to the tubes and rocked on a nutator for 1 h at room temperature. 600 μl of 2% skim milk was added to 200 μl of the rescued pecan126 phage library and rocked for 1 h to block nonspecific phage binding. The blocked phage library aliquot was split into two tubes containing stage 11 and 14 embryo homogenates and rocked for 1 h. These two tubes were centrifuged at 1000 g for 1 min to precipitate the particulate material containing the bound phages, whereas the supernatant was discarded. After washing ten times with PBS and another ten times with PBS with 0.1% Tween 20 (PBS-T), bound phages were eluted with 200 μl of 100 mM triethylamine by rocking for 9 min. After centrifugation at 100 g for 1 min, the supernatant was transferred to tubes that contain 100 μl of 1 M Tris-HCl (pH 7.5) to neutralize the triethylamine. 150 μl of the mixture containing eluted phage was infected into 10 ml of HBV88 cells that had been cultured with Cm to OD_600_ = 0.8. The infection was performed static at 37°C for 30 min. The infected bacteria were collected and spread on a 15 cm plate of 1.5% agar in 2 x YT + Amp and Cm with 10 times dilution or on another plate without dilution to obtain isolated colonies. 40 pools of 20 colonies were made from stage 11 or stage 14 phage display group. Periplasm was prepared from these pools as described below.

To deploy the phage for Western blot membrane probing, lysates from stage 11, 15 and 26 embryos were resolved on 10% SDS-PAGE and transferred to PVDF membrane (1.2 embryo equivalents per lane) as described below [24]. The PVDF membrane was cut into pieces, incubated with 2% skim milk in PBS for blocking for 30 min and washed with PBS three times. 30 μl of the pecan126 phage library was incubated in 5 ml of 2% skim milk in PBS for blocking for 30 min. The phage library mixture was divided into three and added to three pieces of the blocked membrane. After the incubation for 1 h, the membranes were washed with PBS 10 times and subsequently with PBS-T 10 times. Bound phages were eluted with 1.5 ml of 100 mM triethylamine by incubating for 9 min. The eluted phage was neutralized with 0.75 ml of 1 M Tris (pH 7.5). 375 μl of phage eluates from three stages were used to infect 10 ml of HBV88 cells that were grown to OD_600_ = 0.46 in LB + Cm and left static at 37^o^ C for 30 min. The infected bacteria were spread on one 9 cm plate without dilution and another plate after 10-fold dilution to obtain isolated colonies. 20 pools of 10 colonies each were made for each phage preparation.

### Phage display screen of semi-synthetic llama nanobody library

Nomad#1 library consists of hyperdiversified nanobody genes originally isolated from three unimmunized llamas in pecan21 phage display vector that fuses the antibody to M13 gene 3 [25]. Ndel1-MBP consists of the N-terminal 242 amino acids of *Xenopus* Ndel1 fused to maltose binding protein (MBP). LIS1-MBP consists of the N-terminal 90 amino acids of *Xenopus* LIS1 fused to MBP. Both MBP fusions were provided by Victoria J. Allan (University of Manchester). Conventional panning methods were used for these targets [26]. An 8-well strip with high binding capacity (Costar) was coated with 8 x100 μl of 10 μg/ml of Ndel1-MBP or LIS1-MBP proteins overnight at 4°C. The coated wells were washed with PBS three times and blocked with 2% skim milk in PBS for 1 h. 80 μl of Nomad1 phage library (1 x 10^12^ cfu/ml) was diluted 10 times with 2% skim milk in PBS and incubated for 1 hour for blocking. For phage panning using Ndel1-MBP, 800 μl of the diluted phage library was preincubated with 8 μg of LIS1-MBP, while for phage display to LIS1-MBP, the diluted phage library was preincubated with 8 μg of Ndel1-MBP to eliminate anti-MBP binders. 8 x 100 μl of the preincubated phage library was added to 8 wells and incubated on a nutator for 1 h and subsequently static for 1 h. After the incubations, the 8-well strips were washed with PBS-T 10 times and PBS 10 times. 8 x 100 μl of 100 mM triethylamine was added to the 8 well strip and incubated for 10 min for eluting phages. The eluted phages were collected and neutralized with 400 μl of 1 M Tris (pH 7.5). 600 μl of the neutralized phage eluates were infected into 10 ml of mid-exponential phase XL1-Blue in 2 x YT/2% glucose with 30 μg/ml tetracycline (Tet) for 30 min at 37°C. An aliquot of the infected bacteria was serially diluted (1/10, 1/100, 1/1000) and spread on 10 cm plates for titer check and for obtaining single colonies to prepare monoclonal phages. The rest of the infected bacteria were gently pelleted and spread on 15 cm plates. The plates were incubated overnight at 37°C.

Bacterial colonies on 15 cm plates were collected with 3 ml 2 x YT/2% glucose with scraper. 900 μl of the bacterial suspension was mixed with 900 μl of 2 x YT/30% glycerol and stored at -80°C. 800 μl of the suspension was added to 40 ml of 2 x YT/2% glucose with Amp/Tet to obtain an OD_600_ = 0.1 and incubated for 1 h, followed by adding M13K07 helper phage at an MOI of 20 and incubating static for 1 hour at 37 ^o^C. The bacterial culture was further incubated with kanamycin (final 70 μg/ml) and IPTG (1 mM final) overnight on a shaker at 30°C to rescue phage. 2 ml of the culture was centrifuged at 14000 rpm for 1 min and 800 μl of the supernatant containing rescued phage was mixed with 200 μl of 10% skim milk in PBS for the next round of panning. The rest of the phage was kept at -80 ^o^C for polyclonal ELISA. For the second round of panning, 8-well strips were coated with 10 μg/ml of Ndel1-MBP and LIS1-MBP, but washing after phage incubation was performed with PBS-T and PBS (20 times each). For the third and fourth rounds of panning, 8 well strips were coated with 1 μg/ml of Ndel1-MBP and LIS1-MBP and washing was performed with PBS-T and PBS (40 times each).

For monoclonal phage preparation, single colonies with phage display to Ndel1 obtained in titer check plates were picked from round 1 to 4 of panning and cultured in two 96 well plates. A Boekel replicator was used to subculture the clones for small-scale rescue and display while the remainder was made to 15% glycerol and stored frozen.

### ELISA with phages or purified nanobodies

ELISA was performed using high binding 96-well plates coated with 100 μl per well of 1 μg/ml of MBP-Ndel1, MBP-Lis1 or BSA in PBS overnight at 4^o^ C. The coated plates were washed three times with PBS and blocked with 2% skim milk in PBS for 1 h. Polyclonal or monoclonal phage solutions diluted with 2% milk in PBS were added to the plates for 1 h, then washed in PBS-T and PBS (3 times each). After the washes, anti-M13 phage antibody conjugated with HRP (GE Healthcare) at the dilution of 1/5000 with 2% milk in PBS was added to the plate and incubated for 1 h. The washing was repeated, followed by adding 1-Step Ultra TMB-ELISA substrate solution (Thermo Fisher). When signals appeared, 2.5 M sulfuric acid was added to the plates to stop the color reaction. ELISA signals were measured as absorbance ratio 450 nm/ 405 nm in a microplate reader (BioRad model 680).

For ELISA with purified nanobodies, 96 well plate was coated with 1 μg/ml Ndel1-MBP or BSA and blocked as described above. Purified nanobodies as described below were diluted in 2% skim milk in PBS/0.05% Tween-20 at 2 μg/ml and incubated on the coated plate for 4 h at room temperature. After washes with PBS-T, mouse anti-His-tag antibody (1/2000, GeneTex) was added for 1 h at room temperature. The plate was incubated with anti-mouse IgG conjugated with horseradish peroxidase (1/2000, Jackson Immunoresearch) for 1 h. After washes with PBS-T, 1 mg/ml OPD in a solution of 0.1 M citric acid, 0.2 M Na_2_HPO_4_, pH 5, 1 μl/ml H_2_O_2_ was added (140 μl per well) and incubated until color reaction developed. 100 μl of concentrated HCl was added to each well to stop the reaction.

### Nanobody production and purification

Pools or monoclonal colonies harboring pecan126 phagemid clones were cultured in 3–5 ml LB media containing 50 μg/ml Amp until the culture reached OD_600_ = 0.4–0.6. Protein production was induced by adding 0.5 to 1 mM IPTG and cultured for 5–6 h at 37^o^ C or overnight at 30^o^ C. After centrifugation at 13,000 rpm for 2 min, pellets were dissolved in 100 μl of TES buffer (0.1 M Tris-HCl, pH 8.0, 1 mM EDTA, 500 mM sucrose and 1 mM PMSF) and kept on ice for at least 30 min. For osmotic shock, 150 μl of cold 0.2 x TES buffer was added for at least another 30 minutes. Periplasm containing nanobodies was collected in the supernatant after centrifugation at 13000 rpm for 20 min, and NaCl was added to 300 mM final concentration and the extract stored at 4 ^o^C. These periplasm preparations were used for immunostaining and immunoblotting as described below.

12 clones on 96 well plate containing monoclonal bacteria obtained through phage display to Ndel1 were chosen based on positive ELISA signals and cultured in LB +100 μg/ml Amp. After boiling preps of pecan21 plasmids containing nanobody cDNA, DNA sequencing was performed, which revealed that 12 clones were classified into 5 groups. Five plasmids with distinct sequences were digested with *Sfi* I and subcloned into *Sfi* I digested pecan16 vector that encodes His^6^ tagged alkaline phosphatase at the C-terminus [27]. Plasmid DNA was isolated and re-transformed into BL21Star cells and plated. The obtained colonies were cultured in 3 ml of LB + Amp at 37° C until cultures reached the OD of 0.47–0.69. IPTG was added at the concentration of 1 mM to induce protein expression overnight at 30° C. Periplasm was prepared from the culture as described above.

Monoclonal nanobodies were purified from periplasm obtained from 100–500 ml culture through Ni-ion affinity chromatography using His60-Ni-Superflow resin (Clontech) according to the manufacturer’s instructions. Monoclonal nanobodies were eluted from the resin with 50 or 250 μl of 250 mM imidazole buffer (pH 8.0) five times. Total protein level was measured at OD_595_ nm with BioRad protein assay using BSA as standard. The eluates were either dialyzed with PBS or frozen after adding glycerol to 50% and used for immunostaining, immunoblotting and immunoprecipitation as described below.

### Mammalian cell transfection

HEK293T cells (ATCC) were cultured in Dulbecco’s modified Eagle’s medium (DMEM-GIBCO) supplemented with 10% fetal bovine serum (Gemini-Bioscience), when semiconfluent, were transfected with plasmids using linear polyethylenimine (MW 25,000; Polysciences) as described [28]. Briefly, Sport6-Ndel1 DNA (Dharmacon) was diluted in serum free DMEM at 1 μg/μl and mixed with polyethylenimine (1 mg/ml in H_2_O) at 3:1 ratio, the transfection mix was added to the cells for 6 h. The cells were washed with PBS, cultured in serum-containing DMEM for 48 h, then lysed in 1% Triton X100, 50 mM NaCl, 1 mM EDTA, 10 mM Tris HCl (pH 7.5) containing protease inhibitor cocktail (Roche) for protein analysis.

### Immunostaining of cryosections, immunoblotting and immunoprecipitation

For cryosectioning, embryos were devitellinized at stage 10, 11 and 12.5, fixed in MEMFA for 1–2 hours and washed with PBS. The fixed embryos were re-fixed with Dent’s fixative overnight at -20 ^o^C, washed with PBS and embedded in 15% sucrose/15% fish gelatin solution as described [29, 30]. The embedded embryos were frozen on dry ice and sectioned at 10 μm with Leica CM3050 cryostat. Cross-sections of the embryos were probed with periplasm solutions from nanobody pools or monoclonal nanobodies (1/2 with PBS) or purified nanobodies (1–3 μg/ml), followed by anti-His tag mouse monoclonal antibodies (Abcam, 1/1600) and anti-mouse IgG conjugated with Alexa488 (Invitrogen, 1/500). Nuclei were stained with DAPI (1 μg/ml). Images were captured on a AxioImager microscope with AxioVision software (Zeiss).

Western blot analysis was carried out with standard techniques as described [24]. Embryo lysates from 4–8 cell stage, stage 9, 10, 11, 14, 15, 26 containing the equivalent of 0.2–1.2 *Xenopus* embryos or 1/40 of 6-well-plate lysates of transfected HEK-293T cells prepared with 1% Triton-X100 buffer (1% TritonX100, 50 mM TrisHCl, pH 7.5, 1 mM EDTA, 50 mM NaCl) were separated on 8–10% SDS-PAGE and the proteins were transferred to the PVDF membrane. The PVDF membrane was either used for phage display or incubated overnight with a purified His-tagged nanobody (0.2–3 μg/ml) at 4°C. After washing in PBS-T, the membrane was incubated with anti-His^6^ tag antibodies (mouse, Genetex, 1/2000) and HRP-conjugated anti-mouse IgG (Jackson, 1/2000). The signal was developed with enhanced chemiluminescence as described [24]. For alkaline phosphatase (AP) tagged nanobodies from pecan16, Tris-buffered saline with 0.05% Tween20 (TBST) was used throughout immunoblotting. 2 μg/ml of purified nanobodies were used. Staining was developed by AP substrate (Western Blue, Promega S3841).

For pulldown experiments, 2 μg/ml of NbE9 nanobodies were incubated with lysates prepared from 6-well plate of HEK293T cells or 20 embryos at stage 38 in the Triton X100 lysis buffer. For pulldowns with NbG12 or NbT15, 480 embryos from stage 15–19 were lysed and incubated with 300 μl of purified nanobodies (0.7 μg/ml of NbG12 or 0.1 μg/ml of NbT15) for 3 h. 20 μl or 250 μl of His60 Ni Superflow resin (Clontech) was added overnight at 4°C. After washing with lysis buffer, the beads were added to the SDS loading buffer, boiled and the proteins were separated in 8–10% SDS-PAGE, followed by immunoblot analysis with nanobodies and AP substrate or by SimplyBlue (Invitrogen) staining. Gel slices corresponding to the 170 kDa band from the NbG12 and NbT15 (control) pulldowns were subjected to LC-MS/MS analysis by the Keck Laboratory at Yale University.

## Results and discussion

### Sibselection of nanobodies from phage display libraries by immunostaning

To generate a cDNA library encoding nanobodies against *Xenopus antigens*, an adult llama was immunized multiple times with *Xenopus* embryo homogenates, and cDNA was made from RNA isolated from immune llama peripheral blood lymphocytes following established protocols [17, 21]. Nanobody encoding cDNA fragments were amplified with specific nested primers as described [11, 22](see Methods) and subcloned into pecan126 [23]. This phagemid vector, depending on the conditions used, can produce high-levels of nanobody secreted into bacterial periplasm or low levels fused to M13 gene 3 protein that is displayed at the phage surface. The pecan126 cDNA library was electroporated into *Escherichia coli* HBV88 bacteria, capable of expressing an arabinose inducible supE tRNA, to generate a phage display library [23].

**Fig 1:**
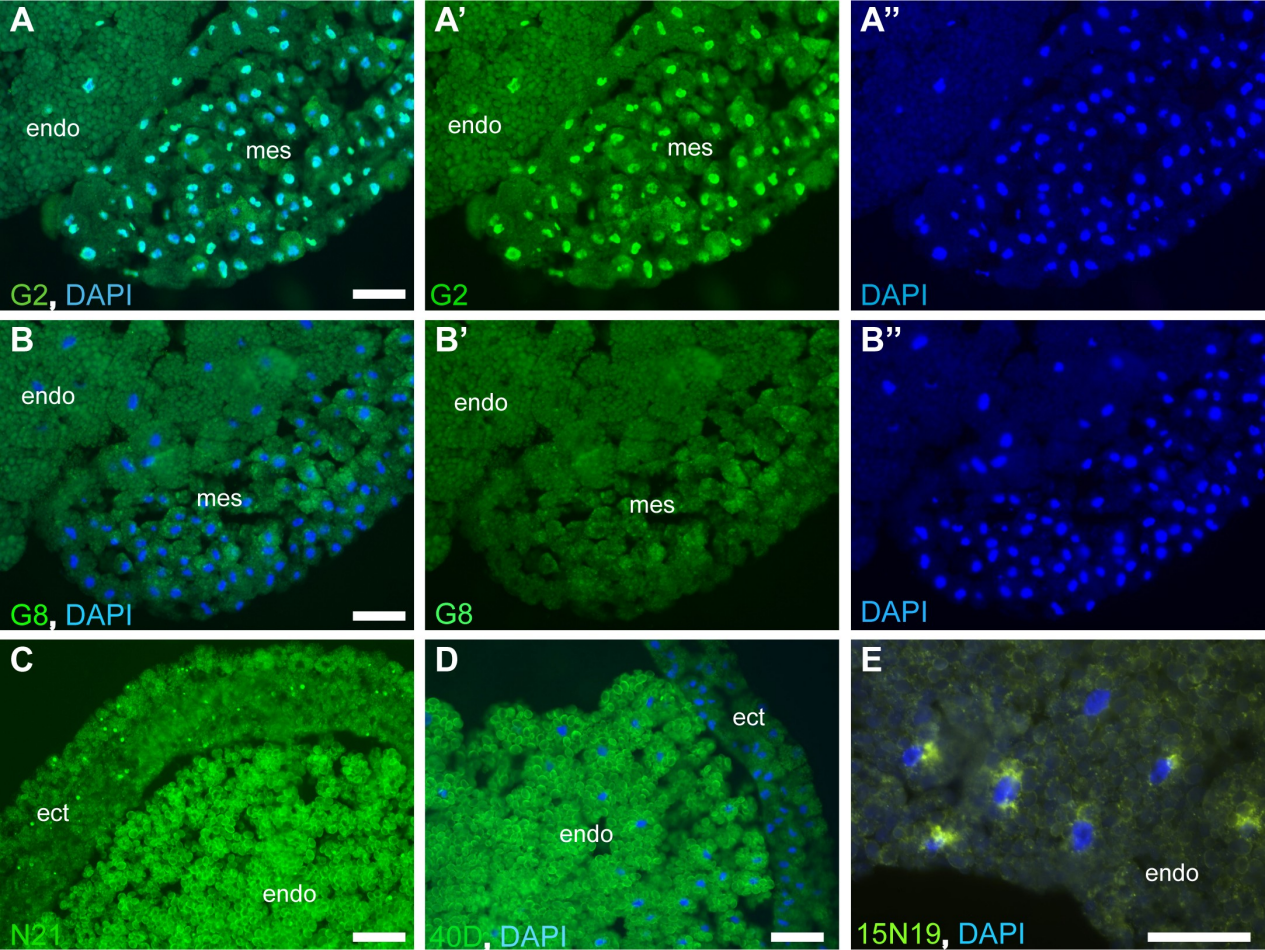
Distinct patterns are revealed by immunostaining of embryo sections with antibody pools. Phages expressing nanobody cDNA library were enriched by binding to immobilized antigens from total embryo homogenates. Periplasm was obtained from pools of 10–20 bacterial colonies infected with enriched phage population, except that pool 15N19 consisted of 3 colonies. Cryosections of stage 11 gastrula embryos were incubated with periplasm pools, followed by mouse anti-His antibodies and Alexa488-conjugated anti-mouse IgG antibodies. (A-A”) Pool G2 stains nuclei; B-B” pool G8 serves as a negative control. (C) Pool N21 reacts with yolk platelets and cytoplasmic puncta. (D) Pool 40D reacts with yolk platelets. (E) Pool 15N19 stains perinuclear Golgi-like structures of endodermal cells. DAPI staining visualizes nuclei (A, A”, B, B”, D, E). Ectoderm (ect), mesoderm (mes) and endoderm (endo) are indicated. Bar, 50 μm.

Phages bearing nanobodies specific to *Xenopus* antigens were enriched by incubating the library with tissue homogenates of fixed gastrula or neurula embryos, or with PVDF membranes blotted with gastrula, neurula and tailbud lysates. The bound phages were eluted with triethylamine, used to infect HBV88 bacteria and rescued with helper phage (see Methods). Periplasm from 10 or 20 bacterial colonies was pooled and used to stain gastrula and neurula tissue sections to deconvolute *Xenopus* reactive nanobodies. A total of 140 periplasmic pools were tested in tissue immunostaining. From these, 17 pools reacted with nuclei, 1 pool with cytoplasmic puncta and yolk platelets, 3 pools with yolk platelets and 3 pools with perinuclear Golgi-like structures (Fig 1). Individual positive nanobodies were obtained after sib-selection; eight clones for nuclear antigens, one for cytoplasmic puncta, two for yolk platelets and three for perinuclear Golgi-like structures.

**Fig 2:**
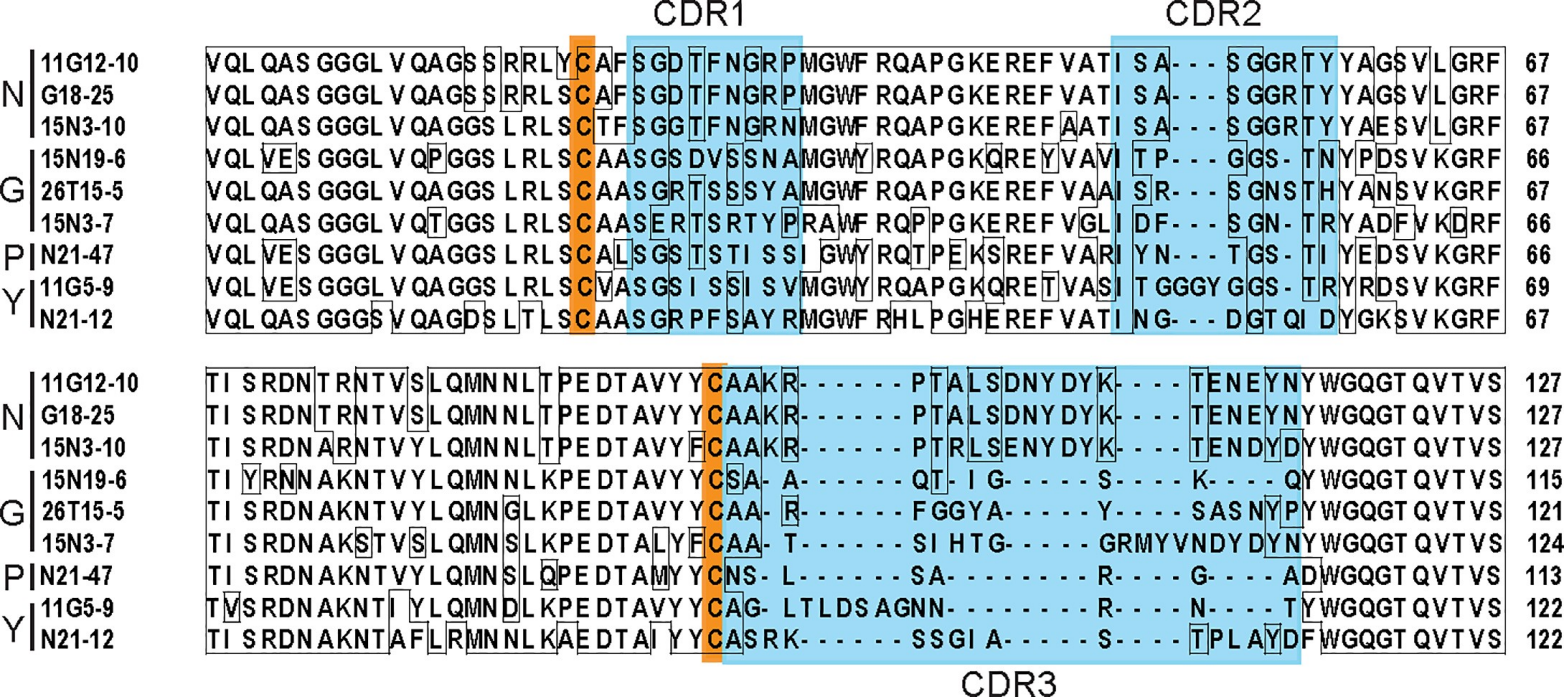
Comparison of unique nanobody amino acid sequences. Nanobodies with different specificities were sequenced and the alignment of deduced amino acid sequences is shown for nanobodies staining nuclei (N), perinuclear Golgi-like structures (G), cytoplasmic puncta (P) and yolk platelets (Y). Complementarity determining regions (CDR1, 2 and 3) are marked by blue boxes, conserved cysteines are indicated as orange boxes.

Plasmid DNAs were prepared from the individual positive clones for sequencing. Eight independently-derived nucleus-specific nanobody cDNAs encoded three distinct amino acid sequences (Fig 2, S2 Fig.). Diverse DNA sequences were found in three clones with perinuclear staining, one clone with punctate staining and two distinct clones with yolk platelet reactivity (Fig 2).

**Fig 3:**
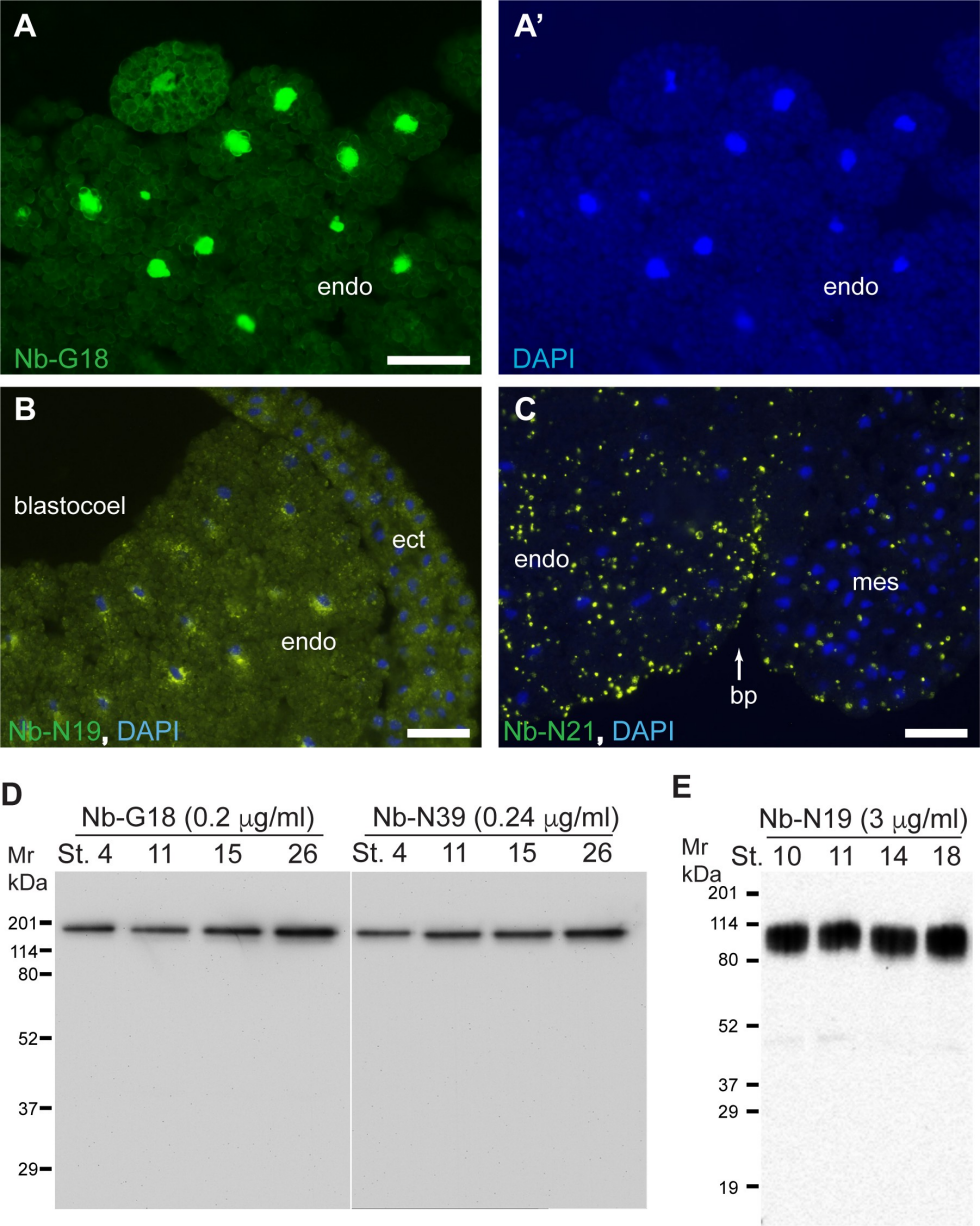
Immunoreactivity of purified nanobodies. Staining of gastrula cryosections with 1.6 μg/ml of NbG18 (A), 3 μg/ml of NbN19 (B) or 1 μg/ml of NbN21 (C) shows nuclear, perinuclear or punctate staining, respectively. DAPI stains nuclei (A’, B, C). Ectoderm (ect), mesoderm (mes), endoderm (endo), blastocoel and blastopore (bp) are indicated. Bars are 50 μm. D-E, immunoblot analysis of embryo lysates at different embryonic stages. Equal amounts of embryo lysates corresponding to the equivalent of 0.5 embryo were separated on 10% SDS-PAGE, transferred to PVDF membrane and probed with different nanobodies. D, NbG18 and NbN39 detect a single protein band of approx. molecular weight of 170 kDa; E, NbN19 detects a broad protein band of 80–95 kDa.

Several candidate nanobodies were purified from periplasmic extracts by immobilized metal affinity chromatography (IMAC) and used for immunostaining of gastrula cryosections and immunoblotting with lysates from different embryonic stages. NbG18 and NbN39, as well as Nb-G12, Nb-G2, stained nuclei and recognized a 170 kDa band by immunoblotting throughout early embryonic stages (Fig 3). Their similar immunochemical properties matched closely related amino acid sequences. NbN19 recognized perinuclear structures predominantly in the endodermal cells at gastrula to neurula stages and detected a specific band around 85–90 kDa by immunoblot analysis. Staining with NbN21 revealed cytoplasmic puncta (Fig 3). We conclude that our immune phage display library represents a good source of embryonic antigen-specific nanobodies that are capable of binding native and non-native proteins (S2 Fig).

### Identification of Npm2 as the antigen recognized by several nanobodies

**Fig 4:**
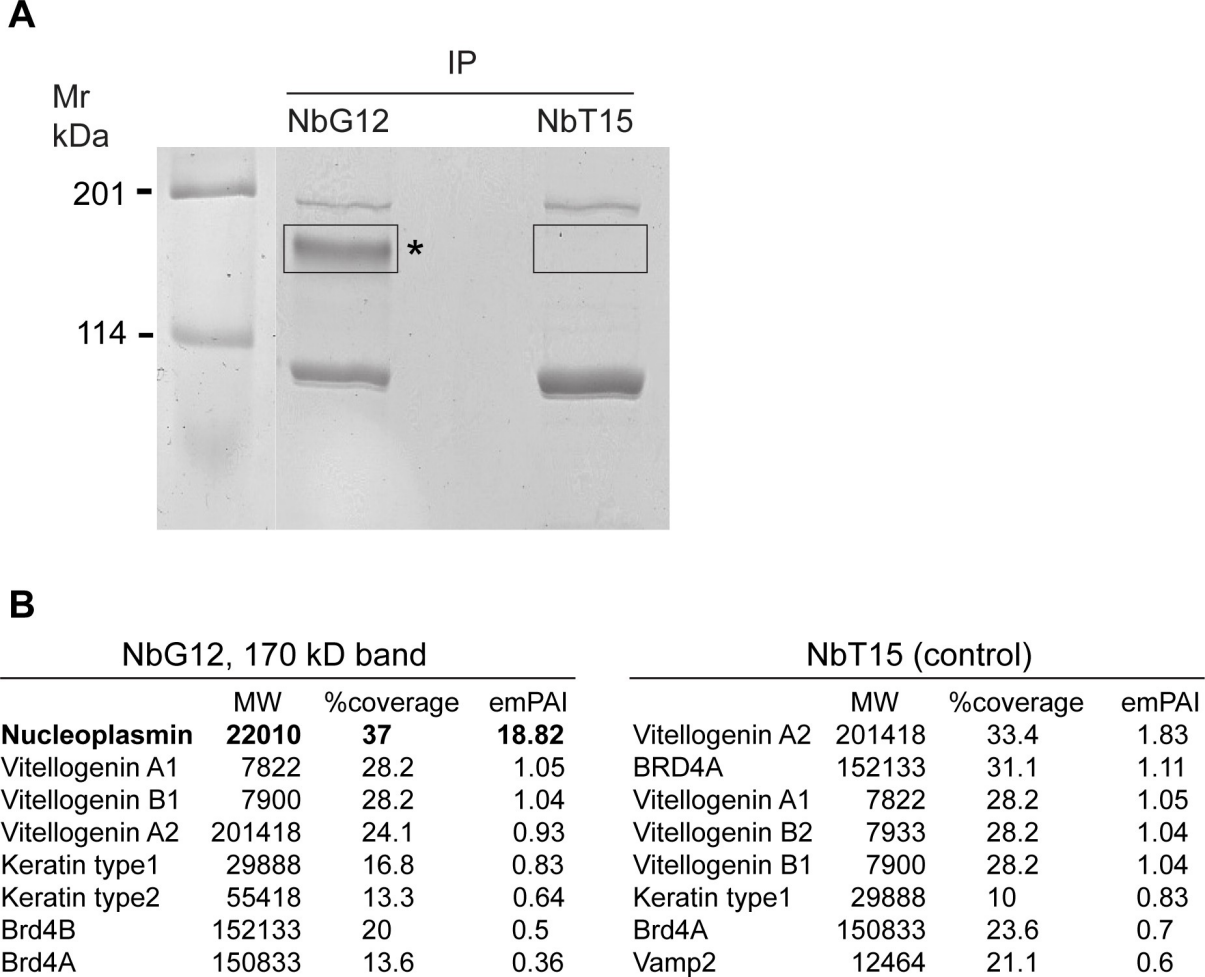
Identification of Npm2 as an antigen recognized by NbG12. (A) Reverse proteomics approach to identify the antigen recognized by NbG12. Lysates from 50 gastrula embryos were precipitated with 7 μg of NbG12 or NbT15 and 25 μl of Ni ion resin and separated by SDS-PAGE. After Simply Blue staining, 170 kDa protein band that was precipitated with NbG12 and the corresponding gel area from NbT15 pulldown were excised for LC-MS/MS analysis. (B) Top hits from the LC-MS/MS analysis of the 170 kDa band. Npm2 is detected with high abundance in NbG12 but not in NbT15 pulldowns.

To identify the nuclear antigen bound by NbG12, we decided to use reverse proteomics. Pulldown of endogenous antigens using NbG12 revealed a prominent Coomassie blue-stained 170 kDa band that corresponds to the band recognized with nuclei-specific nanobodies (Fig 4A, compare with Fig 3D). This band was absent when the pulldown was carried out with NbT15, a nanobody staining perinuclear structures (Fig 4A).

The 170 kDa band was cut out from the gel and subjected to LC-MS/MS analysis. Among top-scored proteins with significant peptide coverage in the experimental but not control fraction was the histone-binding chaperone Npm2 or nucleoplasmin [31–33](Fig 4B). We conclude that Npm2 is the antigen recognized by NbG12 and likely by seven other nanobodies reacting with a band of similar mobility. Although Npm2 has a predicted molecular weight of 22 kDa, it is known to form stable decamer complexes that migrate around 170–180 kDa in SDS-PAGE [34]. We propose that NbG12 recognizes a conformational antigenic determinant that is specific for the Npm2 oligomer rather than the monomer. Alternatively, Npm2 oligomers may represent the preferred state of Npm2 in embryonic cells. The Npm2 determinant appears to be highly immunogenic, because we isolated eight independent nanobodies that stain nuclei on embryo sections and detect a protein band of the same size on immunoblots.

### Isolation of Ndel1-specific nanobodies using a non-immune semi-synthetic llama nanobody library

**Fig 5:**
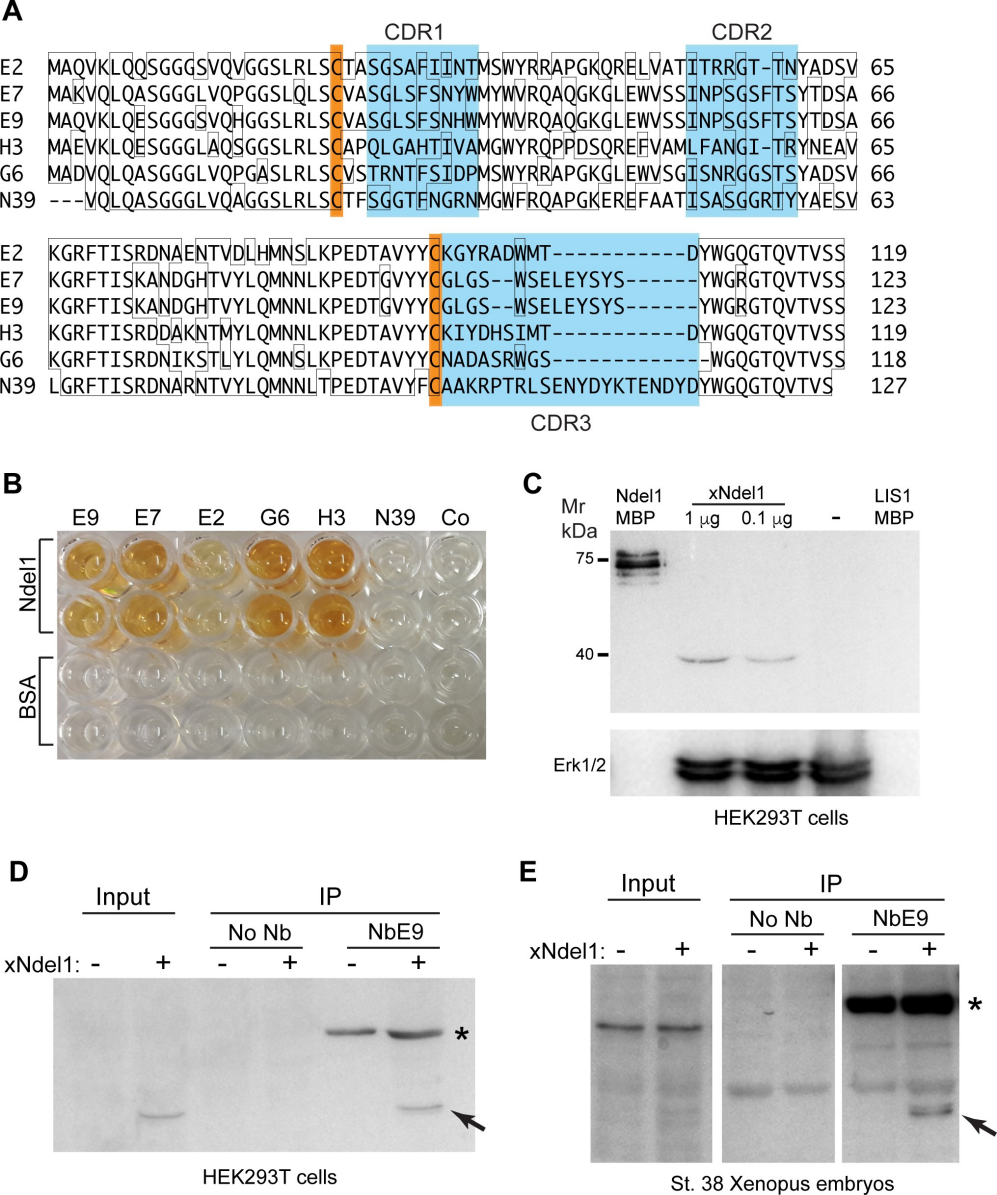
Isolation of Ndel1-specific nanobodies. (A) amino acid sequence alignment of six nanobodies. Orange boxes indicate the two conserved cysteine residues, and blue boxes indicate the three complementarity-determining regions (CDRs). (B) wells coated with purified Ndel1-MBP protein (1 μg/ml) are positive in ELISA after probing with several Ndel1-specific nanobodies but not with negative controls (no Nb Co or NbN39). Control BSA coating confirms specificity. (C) immunoblotting of *Xenopus* Ndel1. Lysates from xNdel1-transfected or control (-) HEK293T cells were separated by SDS-PAGE and visualized with 2 μg/ml of purified AP-NbE7 and AP substrate. Purified Ndel1-MBP, but not LIS1-MBP is detected with NbE7. ERK1/2 antibody controls loading. (D, E) Ndel1 is precipitated by NbE9 from transfected cell lysates (D) and injected *Xenopus* embryos (E). (D) HEK293T cells were transfected with *Xenopus* Ndel1 plasmid as indicated. Cells were lysed after 24–48 h culture. Pulldowns were carried out with 2 μg of 6His-AP-NbE9 (asterisk) and 20 μl of Ni-agarose beads. (E) four-cell embryos were injected with 100 pg of xNdel1 DNA. The injected embryos were lysed at stage 38 and the pulldown was performed as in D. Arrows in D, E, indicate the 39 kD band corresponding to xNdel1. AP-NbE9 and AP substrate were used in immunoblotting for signal detection (D, E).

Because a limited range of nanobodies was identified in the immune llama library, the immune response may have been directed to a few strong immunogens present in *Xenopus* homogenates. We therefore decided to take an alternative approach and used a semi-synthetic non-immune llama nanobody library in pecan21 [25]. As a proof-of-principle, we carried out phage panning on wells coated with two highly purified *Xenopus* antigens Ndel1-MBP and LIS1-MBP [35] [36] [37]. To eliminate anti-MBP binders, the library was preincubated with LIS1-MBP or Ndel1-MBP before applying to wells coated with Ndel1-MBP or LIS1-MBP, respectively. After four rounds of panning and elution, polyclonal phages were tested in ELISA on plates coated with respective antigens. Positive reactivity was observed for Ndel1-MBP-coated wells with virtually no cross-reactivity to LIS1-MBP or BSA. Sequencing revealed five groups of phages with distinct complementarity-determining regions (CDR) that are involved in Ndel1 binding, from which five unique clones were chosen for further analysis (Fig 5A).

### Detection of Ndel1 protein in mammalian cells and Xenopus embryos

The selected inserts from the positive monoclonal phages were subcloned into pecan16 to generate His^6^-tagged alkaline phosphatase protein fusions [27]. The nanobodies were purified from periplasmic extracts using IMAC and used for ELISA and immunoblotting. ELISA confirmed specificity for 5 different antibodies (NbE9, NbE7, NbE2, NbG6, NbH3, Fig 5B) that bound Ndel1 but did not react with BSA (negative control). By contrast, NbN39 that is specific for Npm2 did not bind Ndel1.

We next assessed whether the nanobodies can detect Ndel1 after immunoblotting. HEK293T cells were transfected with a plasmid encoding *Xenopus* Ndel1. NbE7 strongly reacted with purified Ndel1-MBP mixed into the untransfected cell lysate, whereas LIS1-MBP was undetectable in these conditions (Fig 5C). Moreover, a sharp band of the predicted size (39 kDa) appeared in cells transfected with *Xenopus* Ndel1 plasmid (Fig 5C). Collectively, these results demonstrate that NbE7 can detect denatured Ndel1 protein.

To address whether NbE9 can immunoprecipitate Ndel1 protein from cultured mammalian cells, the lysates of HEK293T cells transfected with *Xenopus* Ndel1 expression plasmid were incubated with NbE9 and Ni affinity resin. We found that NbE9 pulled down transfected *Xenopus* Ndel1 (Fig 5D, arrow). Similarly, embryos injected at the 4-cell stage with 100 pg of a plasmid containing *Xenopus* Ndel1 cDNA were lysed at stage 38 and the pulldown was done with NbE9 and Ni agarose beads overnight. E9 pulled down injected *Xenopus* Ndel1 from embryo lysates (Fig 5E, arrow). These results demonstrate that purified nanobodies can precipitate *Xenopus* Ndel1 from transfected cells and injected embryos.

### Llama nanobody phage display libraries as a source of immunoreagents for cell and developmental biology

Our work evaluated immune and non-immune phage display approaches for delivering llama single domain antibodies specific for *Xenopus* embryo components. We successfully identified a range of nanobodies that are not only specific for several *Xenopus* antigens, but are also capable of being employed in a variety of immunological methods. Our results not only serve as a proof-of-principle for this approach, but also allow us to formulate several guidelines for future analyses. First, for immunogenic determinants, we could define the target epitope (Npm2 oligomer) by reverse proteomics, using traditional pulldown combined with mass spectrometry analysis. Second, llama immune response appears to be dominated by ‘strong’ nanobody clones that are specific to a small number of immunogenic epitopes. This issue may have prevented us from isolating nanobodies to specific proteins of interest such as Ndel1 from our immune library. Third, given the success of this and other studies with non-immune llama libraries, the immunization process may be unnecessary for production of *Xenopus* specific nanobodies. Fourth, the antibodies that we characterized appear to work in a variety of assays, from ELISA and immunofluorescence to immunoprecipitation and immunoblotting (S2 Fig). Overall, we conclude that phage display approaches will likely help identify many *Xenopus* embryonic antigen-specific nanobodies needed for cell biological and proteomic experiments vital to advance developmental studies. The identified nanonobodies that are specific for Nucleoplasmin will be useful for future analysis of nuclear organization and chromatin regulation. The obtained nanobodies for Ndel1 should help studies of microtubule dynamics, mitotic spindle assembly and organelle positioning during cell division of neural progenitors and other embryonic cells.

## Supporting information

S1 Fig DNA sequences of nanobodies obtained from the immunized and non-immunized llama cDNA libraries. (PDF)

S2 Fig Selected nanobodies, their origin and activity validation. (PDF)
