# Supplementary material for: Isolation of nanobodies against Xenopus embryonic antigens using immune and non-immune phage display libraries
Source: PLoS One. 2019 May 2;14(5):e0216083. doi: 10.1371/journal.pone.0216083 (PMC6497274; doi:10.1371/journal.pone.0216083)
Supplement: S1 Fig — (PDF) [file pone.0216083.s001.pdf]

S1 Fig. DNA sequences of nanobodies obtained from the immunized and non-immunized llama cDNA libraries

Nanobodies obtained from the immune llama cDNA library

Nb-G12
GTCCAGCTGCAGGCGTCTGGGGGAGGATTGGTGCAGGCTGGGAGCTCTCGGAGACTCTACTGTGCGTTCTCGGGAGATAC
CTTCAATGGCCGTCCCATGGGCTGGTTCCGCCAGGCTCCTGGGAAGGAGCGTGAATTTGTTGCCACTATTAGTGCGAGTGGT
GGTCGCACATACTATGCAGGCTCTGTGCTGGGCCGATTCACCATCTCCAGAGACAACACCAGGAACACGGTGAGTCTGCAAA
TGAACAACCTGACACCTGAGGATACGGCCGTTTATTACTGTGCAGCGAAGAGACCGACAGCACTTAGTGATAATTACGACTAC
AAGACGGAGAATGAATATAATTACTGGGGCCAGGGGACCCAGGTCACCGTCTCC

Nb-G18
GTGCAGCTGCAGGCGTCTGGGGGAGGATTGGTGCAGGCTGGGAGCTCTCGGAGACTCTCCTGTGCGTTCTCGGGAGATAC
CTTCAATGGCCGTCCCATGGGCTGGTTCCGCCAGGCTCCTGGGAAGGAGCGTGAATTTGTTGCCACTATTAGTGCGAGTGGT
GGTCGCACATACTATGCAGGCTCTGTGCTGGGCCGATTCACCATCTCCAGAGACAACACCAGGAACACGGTGAGTCTGCAAA
TGAACAACCTGACACCTGAGGATACGGCCGTTTATTACTGTGCAGCGAAGAGACCGACAGCACTTAGTGATAATTACGACTAC
AAGACGGAGAATGAATATAATTACTGGGGCCAGGGGACCCAGGTCACCGTCTCC

Nb-N39
GTGCAGCTGCAGGCGTCTGGGGGAGGATTGGTGCAGGCTGGGAGCTCTCGGAGACTCTCCTGTGCGTTCTCGGGAGATAC
CTTCAATGGCCGTCCCATGGGCTGGTTCCGCCAGGCTCCTGGGAAGGAGCGTGAATTTGTTGCCACTATTAGTGCGAGTG
GTGGTCGCACATACTATGCAGGCTCTGTGCTGGGCCGATTCACCATCTCCAGAGACAACACCAGGAACACGGTGAGTCTG
CAAATGAACAACCTGACACCTGAGGATACGGCCGTTTATTACTGTGCAGCGAAGAGACCGACAGCACTTAGTGATAATTA
CGACTACAAGACGGAGAATGAATATAATTACTGGGGCCAGGGGACCCAGGTCACCGTCTCC

Nb-G2
GTGCAGCTGCAGGCGTCTGGGGGAGGATTGGTGCAGGCTGGGAGCTCTCGGAGACTCTCCTGTGCGTTCTCGGGAGATAC
CTTCAATGGCCGTCCCATGGGCTGGTTCCGCCAGGCTCCTGGGAAGGAGCGTGAATTTGTTGCCACTATTAGTGCGAGTG
GTGGTCGCACATACTATGCAGGCTCTGTGCTGGGCCGATTCACCATCTCCAGAGACAACACCAGGAACACGGTGAGTCTG
CAAATGAACAACCTGACACCTGAGGATACGGCCGTTTATTACTGTGCAGCGAAGAGACCGACAGCACTTAGTGATAATTA
CGACTACAAGACGGAGAATGAATATAATTACTGGGGCCAGGGGACCCAGGTCACCGTCTCC

Nb-40
GTCCAGCTGCAGGCGTCTGGGGGAGGATTGGTGCAGGCTGGGAGCTCTCGGAGACTCTCCTGTGCGTTCTCGGGAGATAC
CTTCAATGGCCGTCCCATGGGCTGGTTCCGCCAGGCTCCTGGGAAGGAGCGTGAATTTGTTGCCACTATTAGTGCGAGTG
GTGGTCGCACATACTATGCAGGCTCTGTGCTGGGCCGATTCACCATCTCCAGAGACAACACCAGGAACACGGTGAGTCTG
CAAATGAACAACCTGACACCTGAGGATACGGCCGTTTATTACTGTGCAGCGAAGAGACCGACAGCACTTAGTGATAATTA
CGACTACAAGACGGAGAATGAATATAATTACTGGGGCCAGGGGACCCAGGTCACCGTCTCC

Nb-32
GTCCAGCTGCAGGCGTCTGGGGGAGGATTGGTGCAGGCTGGGAGCTCTCGGAGACTCTCCTGTGCGTTCTCGGGAGATAC
CTTCAATGGCCGTCCCATGGGCTGGTTCCGCCAGGCTCCTGGGAAGGAGCGTGAATTTGTTGCCACTATTAGTGCGAGTG
GTGGTCGCACATACTATGCAGGCTCTGTGCTGGGCCGATTCACCATCTCCAGAGACAACACCAGGAACACGGTGAGTCTG
CAAATGAACAACCTGACACCTGAGGATACGGCCGTTTATTACTGTGCAGCGAAGAGACCGACAGCACTTAGTGATAATTA
CGAC

Nb15N310
GTGCAGCTGCAGGCGTCTGGAGGAGGCTTGGTGCAGGCTGGGGGGTCTCTGAGACTCTCCTGTACGTTCTCTGGAGGCAC
CTTCAATGGCCGTAATATGGGCTGGTTCCGCCAGGCTCCTGGGAAGGAGCGTGAATTTGCTGCAACTATTAGTGCGAGTG
GCGGTCGTACATACTATGCAGAGTCCGTTCTGGGCCGATTCACCATCTCCAGAGACAACGCCAGGAACACGGTGTATCTG
CAAATGAACAACCTGACACCTGAGGATACGGCCGTTTATTTCTGTGCAGCAAAGAGACCGACACGGCTTAGTGAGAATTA
CGACTACAAAACGGAGAATGATTATGATTACTGGGGCCAGGGGACCCAGGTCACCGTCTCC

Nb11G57
GTGCAGCTGCAGGCGTCTGGGGGAGGATTGGTGCAGGCTGGGGGCTCTCTGAGACTCTCCTGTACGTTCTCTGGAGGCAC
CTTCAATGGCCGTAATATGGGCTGGTTCCGCCAGGCTCCTGGGAAGGAGCGTGAATTTGCTGCAACTATTAGTGCGAGTG
GCGGTCGTACATACTATGCAGAGTCCGTTCTGGGCCGATTCACCATCTCCAGAGACAACGCCAGGAACACGGTGTATCTG
CAAATGAACAACCTGACACCTGAGGATACGGCCGTTTATTTCTGTGCAGCAAAGAGACCGACACGGCTTAGTGAGAATTA
CGACTACAAAACGGAGAATGATTATGATTACTGGGGCCAGGGGACCCAGGTCACCGTCTCC

Nb-N19
GTGCAGCTGGTGGAGTCTGGGGGAGGCTTGGTTCAGCCAGGGGGGTCTCTGAGACTCTCCTGTGCAGCCTCTGGAAGCGA
CGTCAGTAGTAATGCCATGGGCTGGTACCGCCAGGCTCCAGGAAAGCAGCGCGAGTACGTCGCAGTGATTACTCCTGGTG
GTAGCACAAATTATCCTGATTCCGTGAAGGGCCGATTCACCATCTATAGAAACAACGCCAAGAACACGGTATATCTGCAG
ATGAACAACCTGAAACCTGAAGACACGGCCGTCTATTACTGTAGTGCAGCTCAAACTATTGGCTCCAAACAATACTGGGG
CCAGGGGACCCAGGTCACCGTCTCC

Nb-T15
GTGCAGCTGCAGGCGTCTGGGGGAGGATTGGTGCAGGCTGGGGGCTCTCTGAGACTCTCCTGTGCAGCCTCTGGACGCAC
CTCCAGTAGCTATGCCATGGGCTGGTTCCGGCAGGCTCCAGGGAAGGAGCGTGAGTTTGTAGCAGCTATTAGTCGGAGTG
GTAATAGCACACACTATGCAAACTCCGTGAAGGGCCGATTCACCATCTCCAGAGACAACGCCAAGAACACGGTGTATCTG

CAAATGAACGGCCTGAAACCTGAGGACACGGCCGTTTATTACTGTGCAGCCAGGTTCGGCGGTTACGCCTACAGCGCCTC
CAATTATCCCTACTGGGGCCAGGGGACCCAGGTCACCGTCTCC

Nb-15N37
GTGCAGCTGCAGGCGTCTGGGGGAGGATTGGTGCAGACTGGGGGATCGCTGAGACTCTCCTGTGCAGCCTCTGAACGCAC
CAGTAGAACTTATCCCAGGGCCTGGTTTCGCCAACCTCCAGGGAAGGAGCGTGAATTTGTAGGACTTATTGATTTTAGCG
GTAATACGCGCTATGCAGATTTCGTGAAGGACCGATTCACCATCTCCAGAGACAACGCCAAGAGCACGGTGTCGCTGCAA
ATGAACAGCCTGAAACCTGAGGACACGGCCCTTTATTTCTGTGCAGCAACTTCGATCCATACTGGTGGTAGAATGTATGT
GAACGACTATGACTATAATTATTGGGGCCAGGGGACCCAGGTCACCGTCTCC

Nb-N21
GTGCAGCTGGTGGAGTCTGGGGGAGGCTTGGTGCAGGCTGGGGGGTCTCTGAGACTCTCCTGTGCACTCTCTGGAAGCAC
CAGTACTATCAGTTCCATCGGCTGGTACCGCCAGACTCCAGAGAAGTCGCGCGAGTTCGTCGCGCGTATTTATAATACAG
GAAGTACAATCTATGAAGATTCCGTGAAGGGCCGATTCACCATCTCCAGAGACAACGCCAAGAACACGGTGTATCTGCAA
ATGAACAGCCTGCAACCTGAGGACACGGCCATGTATTACTGTAATTCACTTTCGGCCCGTGGGGCCGACTGGGGCCAAGG
GACCCAGGTCACCGTCTCC

Nb-11G59
GTGCAGCTGGTGGAGTCTGGGGGAGGCTTGGTGCAGGCTGGGGGGTCTCTGAGACTCTCCTGTGTAGCCTCTGGAAGCAT
CAGCAGTATCAGTGTCATGGGCTGGTACCGCCAGGCTCCAGGGAAGCAGCGCGAGACTGTCGCAAGTATTACTGGTGGTG
GGTATGGTGGTAGTACACGCTATCGGGACTCCGTGAAGGGCCGATTCACCGTCTCCAGAGACAACGCCAAGAATACGATA
TATCTGCAAATGAACGACCTGAAACCTGAGGACACAGCCGTCTATTATTGTGCAGGTCTAACACTCGATAGTGCTGGTAA
CAACCGAAACACTTACTGGGGCCAGGGGACCCAGGTCACCGTCTCC

Nb-N21Y
GTCCAGCTGCAGGCGTCTGGGGGAGGATCAGTGCAGGCTGGGGACTCTCTGACACTCTCCTGTGCAGCCTCTGGACGACC
CTTCAGCGCCTACCGGATGGGCTGGTTCCGCCATCTTCCAGGACACGAGCGTGAATTTGTAGCAACTATTAATGGGGATG
GCACCCAAATAGACTACGGAAAGTCCGTGAAGGGCCGATTCACCATCTCGAGAGACAACGCCAAGAACACGGCGTTTCTG
CGAATGAACAACCTGAAAGCTGAGGACACGGCCATTTATTACTGTGCGTCACGTAAAAGTTCGGGGATCGCTTCGACCCC
TCTTGCGTATGACTTCTGGGGCCAGGGGACCCAGGTCACCGTCTCC

Sequences of Ndel1-specific nanobodies

Nb-E2
ATGGCGCAGGTGAAGCTGCAGCAGTCTGGAGGAGGCTCAGTGCAGGTTGGGGGGTCTCTGAGACTCTCCTGTACAGCCTC
TGGAAGTGCCTTCATTATTAATACCATGTCCTGGTACCGCCGGGCTCCAGGGAAGCAGCGCGAGTTGGTCGCAACTATTA
CGAGAAGAGGGACAACAAACTATGCAGACTCCGTGAAGGGCCGATTCACCATCTCCAGAGACAACGCCGAGAACACGGTG
GATCTGCATATGAACAGTCTGAAACCTGAGGACACGGCCGTCTATTACTGTAAGGGATATCGGGCGGATTGGATGACTGA
CTACTGGGGCCAGGGGACCCAGGTCACCGTCTCCTCA

Nb-E7
ATGGCGAAGGTCCAGCTGCAGGCGTCTGGAGGAGGCTTGGTGCAGCCGGGGGGGTCTCTCCAACTCTCCTGTGTAGCCTC
TGGATTGTCCTTCAGTAACTACTGGATGTATTGGGTCCGTCAGGCTCAAGGAAAGGGGCTCGAGTGGGTCTCATCTATTA
ATCCTAGCGGTAGTTTTACATCTTATACAGACTCCGCGAAGGGCCGCTTCACCATCTCCAAAGCCAACGACGGGCACACG
GTGTATTTGCAAATGAACAACTTGAAACCTGAGGACACAGGCGTCTATTATTGTGGATTAGGGTCATGGTCAGAATTGGA
ATATTCCTATAGTTACTGGGGCCGGGGGACCCAGGTCACCGTCTCCTCA

Nb-E9
ATGGCGCAGGTGAAACTGCAGGAGTCAGGGGGAGGCTCGGTGCAGCACGGGGGGTCTCTGAGACTCTCCTGTGTAGCCTC
TGGATTGTCCTTCAGTAACCACTGGATGTATTGGGTCCGTCAGGCTCAAGGAAAGGGGCTCGAGTGGGTCTCATCTATTA
ATCCTAGCGGTAGTTTTACATCTTATACAGACTCCGCGAAGGGCCGATTCACCATCTCCAAAGCCAACGACGGGCACACG
GTGTATTTGCAAATGAACAACTTGAAACCTGAGGACACAGGCGTCTATTATTGTGGATTAGGGTCATGGTCAGAATTGGA
ATATTCCTATAGTTACTGGGGCCGGGGGACCCAGGTCACCGTCTCCTCA

Nb-H3
ATGGCGGAGGTCAAACTGCAGGAGTCAGGGGGAGGCTTGGCACAGTCGGGGGGGTCTCTGAGACTCTCCTGTGCGCCGCA
ATTAGGTGCACACACTATCGTGGCCATGGGTTGGTACCGCCAACCTCCCGATAGTCAGCGCGAATTTGTCGCGATGCTTT
TTGCTAATGGAATTACACGTTACAATGAAGCCGTGAAGGGCCGGTTCACCATCTCCAGAGACGACGCCAAGAACACGATG
TATCTGCAAATGAACAACCTGAAACCTGAGGACACGGCCGTCTATTACTGTAAGATATATGATCACTCAATTATGACTGA
CTACTGGGGCCAGGGGACCCAGGTCACCGTCTCCTCG

Nb-G6
ATGGCGGATGTGCAGCTGCAGGCGTCTGGAGGAGGCTTGGTGCAGCCTGGGGCATCTCTGAGATTGTCCTGTGTAAGTAC
TCGAAATACCTTCAGTATCGATCCCATGTCCTGGTACCGCCGGGCTCCAGGAAAGGGGCTCGAGTGGGTCTCAGGTATTA
GTAATCGTGGTGGTAGCACAAGCTATGCAGACTCCGTGAAGGGCCGATTCACCATTTCCAGAGACAACATCAAGAGTACG
CTGTATCTGCAAATGAACAGTCTGAAACCAGAGGACACAGCCGTCTATTACTGTAATGCAGATGCAAGTAGGTGGGGTTC
CTGGGGCCAGGGGACCCAGGTCACCGTCTCCTCG
