# Supplementary material for: Isolation of nanobodies against Xenopus embryonic antigens using immune and non-immune phage display libraries
Source: PLoS One. 2019 May 2;14(5):e0216083. doi: 10.1371/journal.pone.0216083 (PMC6497274; doi:10.1371/journal.pone.0216083)
Supplement: S2 Fig — (PDF) [file pone.0216083.s002.pdf]

S2 Fig. Selected nanobodies, their origin and activity validation.

Nanobodies obtained from the immunized llama cDNA library

Antibodies	Clone	Pool	Sequence	IF	WB	IP
Nb-G12	11G12-10	11G12	11G12-10	nuclear	170 kD	170 kD
Nb-G18	G18-25	G18	G18-25	nuclear	170 kD	n. d.
Nb-N39	N39-45	N39	G18-25	nuclear	170 kD	n. d.
Nb-G2	11G2-9	11G2	G18-25	nuclear	170 kD	170 kD
Nb-40	40-35-2	40	G18-25	nuclear	170 kD	n. d.
Nb-N32	N32-53	N32	G18-25	nuclear	n. d.	n. d.
Nb-15N310	15N3-10	15N3	15N3-10	nuclear	n. d.	n. d.
Nb-11G57	11G5-7	11G5	15N3-10	nuclear	170 kD	n. d.
Nb-N19	15N19-6	15N19	15N19-6	golgi	85-90 kD	n. d.
Nb-T15	26T15-5	26T15	26T15-5	golgi	no signal	no signal
Nb-15N37	15N3-7	15N3	15N3-7	golgi	n. d.	n. d.
Nb-N21	N21-47	N21	N21-47	puncta	no signal	n. d.
Nb-11G59	11G5-9	11G5	11G5-9	yolk	45 kD	n. d.
Nb-N21Y	N21-12	N21	N21-12	yolk	45 kD	n. d.

Ndel1-specific nanobodies obtained from the non-immune llama cDNA library

Antibodies	Sequence	ELISA with xNdel1-MBP	IF with xNdel1	Immunoblotting with:		IP with xNdel1
				xNdel1	xNdel1-MBP	
Nb-E7	E7	++	neural	39 kD	++	n. d.
Nb-E9	E9	++	neural	39 kD	++	39 kD
Nb-H3	H3	++	no signal	no signal	+	n. d.
Nb-G6	G6	++	neural	no signal	+	n. d.
Nb-E2	E2	+	no signal	no signal	no signal	n. d.

Summary of the origin and reactivity of specific nanobodies presented in Fig. 2 and Fig. 5A.
IF, immunofluorescence; IB, immunoblotting; IP, immunoprecipitation; n. d., not done
